# Physical Activity Consultation Framework for Primary Care Providers

**DOI:** 10.1177/15598276261495518

**Published:** 2026-11-04

**Authors:** Paolo Capodaglio, Luca Cavaggioni

**Affiliations:** 1 Department of Biomedical, Surgical and Dental Sciences, La Statale University, Milan, Italy (P C); 2 UOC Riabilitazione Muscoloscheletrica e Metabolica, 9354 IRCCS Istituto Auxologico Italiano, Milan, Italy (P C); 3 Department of Theoretical and Applied Sciences, eCampus University, Novedrate, Italy (L C); 4 Department of Endocrine and Metabolic Diseases, Obesity Unit - Laboratory of Nutrition and Obesity Research, 9354 IRCCS Istituto Auxologico Italiano, Milan, Italy (L C)

**Keywords:** physical activity, primary care, sedentary behaviour, lifestyle

## Abstract

Physical activity (PA) is vital for managing chronic diseases, yet it remains under-prescribed in primary care. This commentary argues that translating PA guidelines into clinical practice requires a pragmatic, patient-centred approach. Rather than focussing exclusively on traditional **PA** recommendations, primary care **providers** should encourage reducing sedentary behaviour and integrating daily movement through short bouts of activity (“**PA** snacks”). We propose a simple consultation framework to help **primary care providers** deliver personalized advice, set progressive goals, and conduct follow-ups. **Often, primary care providers represent the first healthcare contact** and are uniquely positioned to initiate behavioural change, identify inactive individuals, and refer patients to qualified exercise professionals for long-term management. Integrating brief **PA** counselling into routine visits, combined with multidisciplinary collaboration, may help overcome adherence barriers and establish PA as a sustainable, scalable, and effective clinical intervention.

“Primary care providers are uniquely positioned to provide authoritative advice, identify inactive individuals, and refer them to qualified exercise professionals.”

## Introduction

Physical activity (PA) is widely recognized as one of the most powerful interventions in preventive medicine, yet translating PA guidelines into routine primary care practice remains a global challenge, particularly given the time constraints faced by primary care **providers**. As a multidisciplinary team with expertise in Physical and Rehabilitation Medicine and clinical exercise science, we recognize that these constraints often limit opportunities for comprehensive **PA** counselling, despite the extensive evidence supporting its clinical value. **PA** is frequently relegated to the margins of physician-patient communication, passed among health professionals within a fragmented continuum of care, or delivered merely as a formal statement.

A significant proportion of the global population fails to meet the recommended PA levels of at least 150 **minutes/week** of moderate**-intensity aerobic activity**, or 75 **minutes/week of vigorous-intensity aerobic activity (or an equivalent combination)**, together with two weekly sessions of muscle-strengthening activities involving all major muscle groups.^1^ Nearly one-third of adults worldwide (1.8 billion people) fail to achieve these recommended PA levels, highlighting the global increase in physical inactivity.^2^ Modern lifestyles are highly sedentary, with prolonged sitting occupying much of adults’ waking time and representing a cardiovascular risk factor^3^ independent of overall physical activity levels.

Focussing exclusively on traditional PA recommendations, although evidence-based, has yielded limited population-level impact. If we truly believe **“Exercise is medicine,”**^4^ we must offer practical strategies to reach a broader segment of the population. A key step is to address sedentary behaviour and physical inactivity together by encouraging patients to reduce sitting time and replace it with any form of movement before progressing to more intense **PA**. This approach, reflected in the WHO Guidelines on Physical Activity and Sedentary Behaviour (2020), may be more feasible for patients with chronic pain, physical limitations, multimorbidity, ageing, or obesity. Breaking up sedentary time into short bouts of movement throughout the day is increasingly recognized as beneficial.^5^

In particular, accumulating short bouts of moderate-to-vigorous PA throughout the day, often referred to as “**PA** snacks,” offers a time-efficient strategy to progressively increase activity levels and improve health^6^ consistent with the latest guidelines. These brief (1-5 min) activities can be woven into daily routines, such as fast walking while commuting, stair sprints, or performing housework, gardening, or carrying shopping bags with greater intensity. Because of their brevity and the lack of need for equipment or facilities, “**PA** snacks” directly address one of the main barriers to PA: lack of time.^7^ They may also represent a more realistic option for individuals unable or unwilling to engage in conventional structured **PA**. Supporting this concept, a recent accelerometer-based study of 73.485 UK Biobank participants compared the equivalence of light and moderate PA with 1 minute of vigorous PA for all-cause and cardiovascular mortality, major adverse cardiovascular events, type 2 diabetes, and cancer outcomes.^8^ The findings suggest that 1 minute of vigorous PA provides risk reductions comparable to 4-9 minutes of moderate **PA** and 53-156 minutes of light PA. In this regard, “minimal dose” approaches, which reduce weekly **PA** volume below guideline thresholds, should not be considered equivalent to meeting current **PA** recommendations. Rather, they may represent a pragmatic entry point for inactive individuals when structured **PA** is perceived as unfeasible, encouraging intermittent, lifestyle-embedded, preferred, and self-paced PA, particularly for those with chronic conditions. This approach may act as a Trojan horse to progressively improve lifestyle, raise **PA** levels, and achieve clinically meaningful health benefits at the population level.

Importantly, even PA levels that do not improve cardiorespiratory fitness can still produce significant metabolic effects, including the production of myokines influencing systemic metabolism, immune function, energy balance, glucose regulation, inflammation, and neurotrophic factors supporting neurogenesis, synaptogenesis, angiogenesis, and gliogenesis.^9^

Rather than strict adherence to “old guidelines,” we should explain that health is shaped by the full spectrum of daily activities, sleep, sedentary behaviour, light-intensity movement, and moderate-to-vigorous PA in a deeply interrelated way.^10^ Improving sleep and reducing sedentary time may create more space and energy for PA, reinforcing the **concept that “every move that replaces sedentary time coun**ts.”^1^ A first “minimal dose” prescription can serve as a stepping stone to more structured activity. Primary care **providers** are uniquely positioned to translate PA recommendations into personalized, persuasive, and achievable advice based on patients’ activity level, health status, preferences, and lifestyle. Particular attention should be given to movement quality, ensuring exercises are performed with appropriate technique, control, and awareness to maximize **PA** benefits, reduce injury risk, and support long-term adherence.^11,12^ Compliance with PA prescriptions depends not only on the prescription itself but also on patients’ motivation, confidence, perceived barriers, and opportunities to integrate movement into everyday life. Accordingly, brief counselling may be strengthened by evidence-based behaviour-change strategies, including shared goal setting, action planning, self-monitoring, problem solving, and planned follow-up.^13^

The purpose of this commentary is not to provide a comprehensive behaviour-change framework, but to offer primary care **providers** a persuasive and feasible approach for bringing PA recommendations into patients’ everyday experience.

## From Guidelines to Prescription: a Practical Approach for Primary Care

Insufficient knowledge of PA recommendations has been identified as a barrier preventing physicians from providing **PA** counselling.^14^ During a consultation, PA recommendations may be structured around a few practical steps: (i) interrupt prolonged sitting regularly, (ii) identify opportunities for short bouts of activity during daily routines, (iii) introduce 1-5-minute “**PA** snacks,” (iv) start with a minimal effective dose and progressively increase frequency or duration, (v) prescribe PA with a clear dosage and progression, and (vi) plan follow-up to review adherence, identify barriers, and adapt the prescription. Rather than simply advising patients to exercise more, physicians should translate PA recommendations into practical actions embedded in everyday life (Table 1).

**Table 1.** Overview of Exercise Snack Modalities and Dosage Characteristics.

Outcome	Exercise Description	Sets	Repetitions
Cardiorespiratory fitness	Ascending and descending a flight of stairs (or, alternatively, brisk marching in place)	1	2 minutes (twice daily)
Upper-limb strength	Wall push-ups performed against a wall, followed by elbow flexion exercises using dumbbells or water bottles (biceps curls)	1	30 repetitions for each exercise (twice daily)
Lower-limb strength	Seated knee extensions followed by standing calf raises	1	15 repetitions for each exercise (twice daily)
Static balance	Single-leg stance with alternating lateral swings of the contralateral lower limb	1	30 seconds on each limb (twice daily)

Reframe the goal from “finding time for exercise” to “finding opportunities for activity” throughout the day, identify realistic opportunities for intermittent moderate-to-vigorous PA, and encourage small, progressive increases while respecting proper exercise form. PA should be prescribed, reviewed, and progressively adapted like any other long-term therapy, with clear dosage, achievable goals, and planned follow-up expressed in lay language. For example: “Avoid remaining seated for prolonged periods, add short walks during the day, or climb stairs several times daily, progressively increasing frequency, duration, or intensity according to tolerance.”

## Conclusion

Primary care **providers’** advice is a powerful motivator and influences patients’ lifestyle behaviours.^15^ Primary care consultations may therefore represent an important first step in initiating behavioural change, with **PA** counselling offering a valuable opportunity within routine clinical practice. Primary care **providers** are the focus of this paper because they **often** represent the first point of contact for PA promotion within the healthcare system. Through their daily contact with patients, they are uniquely positioned to provide authoritative advice, identify inactive individuals, and refer them to qualified exercise professionals. Once referred, Kinesiologists or Exercise and Health Scientists can prescribe, deliver, and monitor individualized **PA** interventions according to the patient’s clinical characteristics and functional needs. Physician counselling may thus act as a catalyst for multidisciplinary **PA** management. Incorporating the term “physical activity” into doctor-patient conversations and using accessible, practical examples rather than abstract recommendations may help initiate a continuum of care that, when appropriate, guides patients towards specialized exercise professionals. However, opportunities for PA counselling in primary care may be limited,^16,17^ and influenced by several factors, including varying levels of PA knowledge and physicians’ own lifestyle habits.^18,19^ Brief counselling may therefore be viewed as the beginning of a longitudinal process, reinforced through follow-up, simple behaviour-change strategies, and multidisciplinary collaboration when appropriate. Recognizing PA as a sustainable, effective, and scalable form of medicine requires it to be recommended with the same accuracy and care as pharmacological treatments. Particularly in resource-constrained settings, complementing traditional facility-based **PA** with scalable, community-based strategies, including “**PA** snacks,” may help overcome major barriers to adherence and progressively increase PA levels.
